# Age-Friendly Research: promoting inclusion of older adults in clinical and translational research

**DOI:** 10.1017/cts.2023.627

**Published:** 2023-09-04

**Authors:** Bryanna De Lima, Allison Lindauer, Elizabeth Eckstrom

**Affiliations:** 1 Division of General Internal Medicine & Geriatrics, Oregon Health & Science University, Portland, OR, USA; 2 Oregon Alzheimer’s Disease Research Center, Oregon Health & Science University, Portland, OR, USA

**Keywords:** Researcher education, barriers, clinical trials, inclusion, older adults

## Abstract

**Introduction:**

Older adults have a high disease burden but are often underrepresented in research studies due to recruitment and retention obstacles, among others. Geriatric research specialists have identified solutions to these challenges and designed frameworks to help other researchers. Our team utilized three frameworks to create an interactive webinar series aimed to educate research team members on Age-Friendly practices.

**Methods:**

We recruited 40 non-aging-trained research team members to participate in a six-session, real-time webinar series from October to November 2022. Sessions were comprised of 20–30 minute didactics and 30–40 minute group discussions. Participants completed pre- and post-program surveys, commitment to change forms, and post-webinar session surveys. Responses were examined for strengths and areas for improvement. Wilcoxon signed-rank tests assessed differences in confidence scores.

**Results:**

Self-reported confidence scores improved after the webinar series. Most participants provided positive feedback and high likeliness to use what they learned and recommend the webinar to others. The strengths were practical tips, applicable tools, and real-world examples. The major area for improvement was information on industry-sponsored trials. The commitment to change responses varied from pledging to use more inclusive language to adapting materials to improve the consent process.

**Conclusion:**

This interactive Age-Friendly Research webinar series was feasible and well received by participants. We created an Age-Friendly Research community fostering commitment to change clinical and translational research to be more inclusive of older adults. Future work will include more information on industry-sponsored trials and expand to other research centers.

## Introduction

Older adults represent a high proportion of disease burden and greatly benefit from research advances [1]. Yet older adult participation in clinical and translational research has been limited [2–4]. Studies often underrepresent older adults due to arbitrary age limits for study inclusion, not enrolling sufficient older adults, or including healthier older adults than the general population [5]. Barriers for this population include multi-morbidity, ageism, transportation needs, mobility restrictions, lack of insurance, communication issues (hearing loss, visual loss, and difficulty understanding complex study protocols), perceived technology constraints, and distrust of research [1,5]. These barriers pose challenges to study recruitment, adherence, retention, and data collection [1]. Comorbidities and concomitant medication and device use may introduce confounding, effect modification, or bias to complicate data interpretation, sample size requirements, and statistical analysis [6].

While the complexities of including older persons are real, involvement of those for whom trial treatments are intended is critical. The US Food and Drug Administration stated in 1989: “patients included in clinical studies should, in general, reflect the population that will receive the drug when it is marketed…There is no good basis for the exclusion of patients on the basis of advanced age alone, or because of the presence of any concomitant illness or medication” [7]. To address such discrepancies, and to ensure inclusion of older adults in research, the National Institute of Health (NIH) implemented the “Inclusion Across the Lifespan” policy (NOT-OD-18-116), which became mandatory in January 2019. This policy requires that investigators submitting human subjects applications to the NIH “address plans for including individuals across the lifespan…so that knowledge gained from NIH-funded research is applicable to all those affected by the researched diseases/conditions” [8]. Exclusion of older adults in clinical and translational research limits generalizability and may lead to inappropriate or harmful recommendations for this vulnerable population [9].

In December 2022, legislation passed in the USA to improve clinical trial diversity that included older adults as an underrepresented group [10]. Sponsors and/or investigators will now need to submit plans to increase enrollment of underrepresented groups in their trial design. Strategies are needed to represent older adults appropriately in clinical and translational research, including adapting recruitment, consent, protocols and/or planned assessments to accommodate cognitive, physical, and logistical issues for older adults [5]. Some strategies have been identified including thoughtful choice of location, flexible scheduling, use of visualization and accessible communication, and building good relationships, but further research is needed on how to involve older adults facing barriers to participation [11].

Specialists in geriatric research have overcome many of these challenges and designed frameworks to help other research teams. In particular, three frameworks have been developed to enhance inclusion of older adults in research. The first was from The Inclusion of Older Adults Working Group through the Integration Across the Lifespan Enterprise Committee (an over-arching committee for all of the Clinical and Translational Research Award [CTSA] hubs supported by the Center for Leading Innovation and Collaboration) [12]. They developed a comprehensive slide set to train research teams who do not have specific geriatric expertise to enhance inclusion of older adults in their studies. Some included topics are “You Should be Recruiting Older Adults,” “Educating Research Teams to meet the NIH Lifespan Inclusion Policy,” “Addressing Common Statistical Issues,” “Community-Engaged Research with Diverse Older Adults,” and “Gerontologized Measures.” Researchers from Duke University and Emory University developed the second framework called the “5Ts” Framework (Target Population, Team, Tools, Time, and Tips to Accommodate) for communicating challenges to inclusion of older adults in clinical research for non-geriatrics-trained research team members [13]. The John A. Hartford Foundation and the Institute for Healthcare Improvement created the final framework, the Age-Friendly Health Systems (AFHS) initiative, to provide evidence-based practices to minimize healthcare-related harm in older adults. The initiative focuses on four core elements of healthcare known as the “4Ms:” What Matters, Medication, Mentation, and Mobility [14]. The 4Ms are interconnected in a way to align care with what matters to the patient and improve care outcomes [15]. We incorporated these three frameworks into a six-session webinar series pilot study aiming to enhance the inclusion of older adults in research so that study participant demographics match disease demographics for all diseases common in older adults. The study’s aims were to 1) educate research team members at our institution on Age-Friendly Research practices and 2) pilot test Age-Friendly Research tools to improve older adult recruitment and retention. The bigger goal of this work is to create Age-Friendly Research champions, teams, tools, culture, and engagement across all studies at our institution and beyond. This paper reports on results of the webinar series.

## Materials and methods

### Setting and participants

Non-aging-trained research team members at our institution who study diseases common in older adults (cancer, neurologic conditions, etc.) were recruited to participate in this prospective pilot study. Our team reached out to potential individuals through personal connections and institution-wide sources such as research-specific newsletters and networks. Interested participants were invited to email our team and indicate which aim(s) they wanted to complete. Participants interested in our first aim, the webinar series, needed to be available during the scheduled Tuesday morning sessions and agree to complete online REDCap surveys. All participants provided written informed consent, and the study was approved by our Institutional Review Board (#24539).

### Intervention and implementation

Our team developed six, 60-minute interactive webinar sessions focused on enhancing research member expertise in recruitment, retention, and analysis of older adults (Table 1). Sessions comprised of 20–30 minute didactics and 30–40 minute group discussions. The webinar series occurred weekly from October to November 2022. Participants were asked to attend the live sessions, participate in group discussions, and complete online surveys following each session. Upon completion of the webinar series and all required surveys, participants received $1000 and an “Age-Friendly Researcher” certificate to acknowledge their achievement. Webinars were recorded for research purposes and accessible by participants for future use.

**Table 1.** Webinar session topics

#	Topic	Overview
1	Age-Friendly Research: What is it and Why Does it Matter?	Introduce Age-Friendly Research, explain the importance of including older adults in research, and provide inclusive communication tips
2	How to design an Age-Friendly Research Study	Learn how to use the 5Ts framework to design an Age-Friendly study
3	Age-Friendly assessments and outcome measures	Utilize the 4Ms framework as a model to guide Age-Friendly measures and share ways to encourage Age-Friendly Research in industry-sponsored trials
4	Including “vulnerable” older adults in research	Discuss the informed consent process and how to consent a legally authorized representative for persons with cognitive impairment
5	Recruiting older adults into research	Identify and address common barriers to including older adults in research and learn how to adapt recruitment materials to be more inclusive.
6	Engaging and retaining older adults participants with cognitive impairment in research	Recognize signs of mild cognitive impairment and dementia, learn how to work with these participants, and learn strategies to retain them in your study

### Data collection and analysis

All participants completed pre- and post-program surveys with self-reported confidence scores in recruiting and retaining older adults in their studies, adapting materials and methods for older adults, and engaging older adults in future research studies (Supplemental File 1). Nonparametric Wilcoxon signed-rank tests assessed differences in confidence scores between time points. Post-program surveys also asked participants for key takeaways and improvements for the webinar series and to rate the webinar series in certain domains. Additionally, participants completed post-session REDCap surveys and commitment to change forms (Supplemental File 2). We reviewed these REDCap surveys to identify strengths and areas for improvement for individual sessions through thematic analysis. R version 4.1.3 (R Foundation for Statistical Computing, Vienna, Austria) was used for statistical analyses.

## Results

Fifty-three interested individuals contacted our team to participate in this research study. Ten research team members were unable to attend the live webinar sessions and/or did not have the ability to test out the Age-Friendly Research tools. Our final sample size was 43 individuals with 40 individuals participating in the webinar portion of the project. Participants were predominately from Cancer (62.5%), Neurology (30%), and Dermatology (5%) departments with varying positions, including professors (5%), clinical research coordinators (32.5%), data managers (10%), and clinical research assistants (22.5%).

### Pre- and post-program surveys

**Figure 1.**
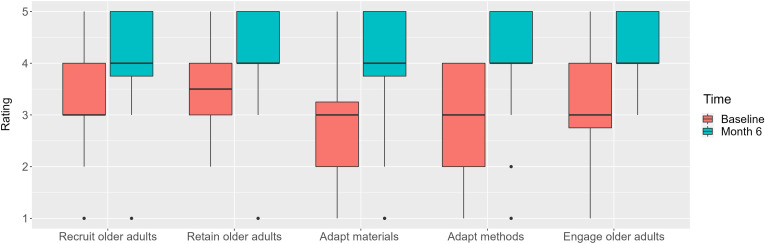
Box plots of self-reported confidence ratings before and after the webinar series on a scale of 1 (not at all confident) to 5 (very confident) for *n* = 40 participants.

Self-reported confidence levels significantly improved across all categories (Fig. 1). On a scale of 1–10 with 10 indicating the highest score, the average rating was 8.2 for the helpfulness of the webinar series, 8.6 for the likeliness of utilizing what they learned, and 8.8 for the likeliness to recommend the webinar to others. Participants’ comments included “The main takeaway I’ve gained from this series is how I can better communicate and facilitate older adults in research,” “small actions on the part of the research team can have a big impact on older adults’ comfort and willingness to participate in research,” and “the real-world examples of what has occurred for others and also what they learned from the experience, and even the real-time ideas other teams had and shared were really helpful and inspiring.” The major critique received was to include more information on industry-sponsored clinical trials.

### Post-session surveys

Feedback was positive: 93.8% reported sessions delivered valuable content and 96.7% stated session objectives were met. About 89% reported high session satisfaction and 80% reported relevance to their research goals. Participants appreciated the communication and practical tips, tools, information on working with cognitively impaired participants, case studies, and discussion time to learn from others’ experiences. Several reported making immediate changes to their project (e.g., buying a pocket talker and increasing font size on materials). The main suggested improvements for the sessions were to provide more real-world examples, offer breakout groups for discussion, and address industry-sponsored clinical trials more.

### Commitment to change

A wide range of commitment to change responses were shared following each webinar session (Table 2). Six general themes emerged: use more Age-Friendly language and materials, create a safe research environment, incorporate the 5T framework, check consent knowledge and involve a legally authorized representative (LAR), listen to and advocate for older adults, and broaden inclusion/exclusion criteria.

**Table 2.** Themes and representative quotes from commitment to change

Theme	Representative Quotes
Use more Age-Friendly language and materials	*“Implement better verbal communication and try to use more than just my words to create a safe and open space to talk about research.”* *“Review recruitment materials to see if the images reflect the sort of diversity we are hoping to see in our study population.”* *“Be more careful on my communication about & with older adults, paying attention to font size of the study materials to ensure participants do not have any difficulty with reading the materials.”* *“Revisit our recruitment materials for plain language and inclusive pictures.”*
Create a safe research environment	*“Ensuring that I’m fostering an environment for our patients to feel comfortable and confident in clinical trial participation, by changing the way we communicate and approach older adults.”* *“Create an environment that will foster open communication, positivity, and inclusivity. I never want my patients to feel like they can't enroll in a study due to any barriers (ex: time, exhaustion, hunger).”* *“Improve my ability to communicate with older adults and improve the comfort of older adults in my research and life.”*
Incorporate the 5T framework	*“Be more mindful when starting new studies that we are designing age-friend[ly] research by ensuring use of the 5Ts, most importantly – Tips to accommodate and Time.”* *“Continue to explore ways to include the older adults into the studies I work with, monitor how I interact with the adults, remember the 5Ts.”*
Check consent knowledge and involve a legally authorized representative (LAR)	*“Begin implementing consent form comprehension questions as part of my informed consent process.”* *“I will go over the slides & information on assent, learn the necessary language, and utilize post-consent quiz.”* “*Aim to be a more ethical researcher and increase the accessibility of my research consent for “vulnerable.” participants and legally authorized representatives. Focusing on simplicity, accessibility, and continuity of consent.”*
Listen and advocate for older adults	*“Turn to my older patients to get insight about what their needs are and make sure I factor in extra time to make sure I am listening to them and trying to relate to them when they don't understand things like technology, terminology, etc.”* *“Advocate more for my patients by requesting special reading material and tools to support recruiting older adults.”* *“Be more aware of the needs of our participants.”* *“Think carefully for transportation methods to make it easier for participants to travel to/from study sites.”*
Broaden inclusion/exclusion criteria	*“Continue to encourage broader inclusion/exclusion in industry clinical trials.”* *“I know now that the FDA prevents exclusion of older adults, and I think having that information will help me be more direct about what our site needs to include them.”* *“I’d like to meet with the clinical investigator on our study to see where we could safely relax some of the exclusion criteria.”* *“Pay attention to the age limits in inclusion/exclusion criteria for our studies and try to discuss with our PIs about talking with sponsors about how we can possibly raise this age to allow for more older adults in our studies.”*

Age-Friendly language and visual adaptations were frequently highlighted. Participants stated that they would “implement better verbal communication” and “revisit recruitment materials for plain language and inclusive pictures.” One participant committed to: “Going forward, I will ensure that all study recruitment materials include at least one image of older adults.”

Participants expressed commitment to incorporating the 5T framework into their research projects. Statements included: “be more mindful when starting new studies that we are designing age-friendly research by ensuring use of the 5Ts, most importantly- Tips to accommodate and Time,” and “Continue to explore ways to include older adults into the studies I work with, monitor how I interact with the adults, remember the 5 Ts.”

Participants also committed to incorporating a consent knowledge check during the consent process and involving a LAR to allow cognitively impaired participants into their study. Representative statements included: “…[I will] learn more about assent and decision-making capacity, as these were topics fairly new to me and I would like to learn more” and “I will make the consenting process more interactive - including asking some of those cognitive check questions throughout the process - regardless of participant age.”

Others emphasized the importance of paying attention to the needs of the participant and what matters to them and advocating for transportation assistance to older study participants who are unable or hesitant to provider their own transportation. One participant wrote that their commitment to change was “ensuring that I’m fostering an environment for our patients to feel comfortable and confident in clinical trial participation, by changing the way we communicate and approach older adults.”

A representative quote of the overall webinar series was: “I pledge to incorporate more person-centered topics in my communication with participants (asking what matters, perhaps asking questions about their support system). I pledge to reflect on the reasons we are excluding older adults from our studies, and how we can learn from it. Our lab bought a pocket talker to better accommodate a hard of hearing older adult in a clinic setting. I was satisfied with this outcome. Change is happening as a result of this seminar!”

## Discussion

This pilot webinar series successfully reached 40 non-aging-trained research team members, improved self-reported confidence, and fostered commitment to transforming their research projects and teams into Age-Friendly Research teams. Attendance at the webinar sessions was excellent (87.3%), and interaction was robust – most weeks we had to cut the discussion short when the hour was finished. Participants reported changes they had made to projects based on prior webinars and reported strong motivation to keep up their Age-Friendly efforts. They found the webinar series to be feasible to incorporate into their workflow and requested recordings to reference in the future.

To our knowledge, this is the first study that combined the 5T framework with the AFHS model to create an Age-Friendly Research model. We provided succinct materials with concrete examples and tools to make it as easy as possible for participants to start making their research more Age-Friendly and were thrilled at the overwhelmingly positive responses we received. Indeed, this pilot study had a goal of recruiting 18 participants and 40 people completed the study! This could be an important first step toward transforming all clinical and translational research into Age-Friendly Research.

One study limitation that participants mentioned frequently was the paucity of information we provided for industry-sponsored trials. Based on early feedback, we added a section to a later webinar on this topic, but it was clear participants wanted more guidance on interacting with industry-sponsored trials. We did get some positive feedback that we had begun to broach this topic, as noted by a participant: “I know now that the FDA prevents exclusion of older adults, and I think having that information will help me be more direct about what our site needs to include them.” “Maybe we can start to push back on the sponsor and let them know we won't provide them if they do not fit age friendly standards.” Another study limitation was that this webinar occurred at just one institution with participants primarily in supporting research roles from two departments. These two departments conduct a large portion of our institution’s clinical and translational research studies though so were ideal candidates for our pilot study. Finally, we did not include objective measures of long-term changes to study protocols or increased inclusion of older adults in research. Larger studies will need to add these important outcomes to their research.

## Conclusion

In summary, this feasible, interactive approach to training research teams to be more Age-Friendly has strong potential for dissemination and broadening the cadre of research members who have added expertise in inclusion of older adults across multiple fields of clinical and translational research. The authors of this paper would be happy to work with anyone interested in conducting a similar training webinar series at their own institution.

## Supporting information

De Lima et al. supplementary material 1 De Lima et al. supplementary material

De Lima et al. supplementary material 2 De Lima et al. supplementary material

## Supplementary material

The supplementary material for this article can be found at https://doi.org/10.1017/cts.2023.627
