# Supplementary material for: Age-Friendly Research: promoting inclusion of older adults in clinical and translational research
Source: J Clin Transl Sci. 2023 Sep 4;7(1):e200. doi: 10.1017/cts.2023.627 (PMC10565191; doi:10.1017/cts.2023.627)
Supplement: De Lima et al. supplementary material 1 — De Lima et al. supplementary material [file S2059866123006271sup001.pdf]

Age-Friendly Research Pre-Program Survey

Please complete the pre-program survey below.

If you have any questions or concerns, please contact Bryanna De Lima at delimab@ohsu.edu.

Age-Friendly Research Team

1) What are the most significant barriers you foresee in enhancing inclusion of older adults in your research?

2) What are the most significant facilitators you foresee in enhancing inclusion of older adults in your research?

Please rate how confident you are in your ability to:

	Not at all	A little bit	Somewhat	Quite a bit	Very much
3) Recruit older adults into your research study?	○	○	○	○	○
4) Retain older adults in your research study?	○	○	○	○	○
5) Adapt documents/forms to accommodate older adults?	○	○	○	○	○
6) Adapt methods to accommodate older adults?	○	○	○	○	○
7) Engage older adults in future research studies?	○	○	○	○	○

Post-Program Survey

Please complete the post-program survey below.

If you have any questions or concerns, please contact Bryanna De LIma at delimab@ohsu.edu.

Age-Friendly Research Team

What are the most significant barriers you foresee in enhancing inclusion of older adults in your research?

What are the most significant facilitators you foresee in enhancing inclusion of older adults in your research?

Please rate how confident you are in your ability to:

	Not at all	A little bit	Somewhat	Quite a bit	Very much
Recruit older adults into your research study?	○	○	○	○	○
Retain older adults in your research study?	○	○	○	○	○
Adapt documents/forms to accommodate older adults?	○	○	○	○	○
Adapt methods to accommodate older adults?	○	○	○	○	○
Engage older adults in future research studies?	○	○	○	○	○

What are some takeaways from this webinar series?

What could be improved from this webinar series?

On a scale of 1-10 (1: lowest, 10: highest), how helpful was the webinar series?

1 10

(Place a mark on the scale above)

On a scale of 1-10 (1: lowest, 10: highest), how likely are you to recommend this webinar series to colleagues?

1 10

(Place a mark on the scale above)

On a scale of 1-10 (1: lowest, 10: highest), how feasible do you think it would be for other researchers to participate in this webinar series?

1 10

(Place a mark on the scale above)

On a scale of 1-10 (1: lowest, 10: highest), how likely are you to utilize what you learned to enhance inclusion of older adults in your research?

1 10

(Place a mark on the scale above)

Please share any other suggestions or comments you have on our webinar series.
