# Supplementary material for: Age-Friendly Research: promoting inclusion of older adults in clinical and translational research
Source: J Clin Transl Sci. 2023 Sep 4;7(1):e200. doi: 10.1017/cts.2023.627 (PMC10565191; doi:10.1017/cts.2023.627)
Supplement: De Lima et al. supplementary material 2 — De Lima et al. supplementary material [file S2059866123006271sup002.pdf]

Age-Friendly Research Session Survey

Thank you for attending this Age-Friendly Research session. Please complete the brief survey below.

Your responses are confidential. Research staff will use an ID number to link your responses over the course of the pilot study, but your name will be not associated with any responses.

You can save the survey and return to it later if needed.

-Age-Friendly Research Team

delimab@ohsu.edu

Please answer the questions below regarding your experience with this session.

Please rate how much you agree with the following statements:

	Strongly disagree	Disagree	Neither agree nor disagree	Agree	Strongly agree
The session delivered valuable content.	◯	◯	◯	◯	◯
The session was well-organized.	◯	◯	◯	◯	◯
There were ample opportunities during the session to ask questions.	◯	◯	◯	◯	◯
The session was relevant to my research goals	◯	◯	◯	◯	◯
The objectives of the session were met	◯	◯	◯	◯	◯

	Too slow	A little too slow	Just right	A little too fast	Too fast
How would you rate the pace of the session?	◯	◯	◯	◯	◯

	Very dissatisfied	Dissatisfied	Unsure	Satisfied	Very satisfied
How would you rate your satisfaction with today's session?	◯	◯	◯	◯	◯

Do you plan to make any changes to your research as a result of this session?

◯ Yes
◯ No

What changes do you plan to make?

What did you like BEST about this session?

What aspects of this session could be improved?

Commitment to Change

As a result of this session I will...

I anticipate the following difficulties or barriers to this change...
